# The orientation of transcription factor binding site motifs in gene promoter regions: does it matter?

**DOI:** 10.1186/s12864-016-2549-x

**Published:** 2016-03-03

**Authors:** Monika Lis, Dirk Walther

**Affiliations:** Max Planck Institute for Molecular Plant Physiology, Am Mühlenberg 1, 14476 Potsdam-Golm, Germany

**Keywords:** Cis-regulatory elements, Transcription factors, Transcription factor binding sites, Motifs, Gene expression

## Abstract

**Background:**

Gene expression is to large degree regulated by the specific binding of protein transcription factors to cis-regulatory transcription factor binding sites in gene promoter regions. Despite the identification of hundreds of binding site sequence motifs, the question as to whether motif orientation matters with regard to the gene expression regulation of the respective downstream genes appears surprisingly underinvestigated.

**Results:**

We pursued a statistical approach by probing 293 reported non-palindromic transcription factor binding site and ten core promoter motifs in *Arabidopsis thaliana* for evidence of any relevance of motif orientation based on mapping statistics and effects on the co-regulation of gene expression of the respective downstream genes. Although positional intervals closer to the transcription start site (TSS) were found with increased frequencies of motifs exhibiting orientation preference, a corresponding effect with regard to gene expression regulation as evidenced by increased co-expression of genes harboring the favored orientation in their upstream sequence could not be established. Furthermore, we identified an intrinsic orientational asymmetry of sequence regions close to the TSS as the likely source of the identified motif orientation preferences. By contrast, motif presence irrespective of orientation was found associated with pronounced effects on gene expression co-regulation validating the pursued approach. Inspecting motif pairs revealed statistically preferred orientational arrangements, but no consistent effect with regard to arrangement-dependent gene expression regulation was evident.

**Conclusions:**

Our results suggest that for the motifs considered here, either no specific orientation rendering them functional across all their instances exists with orientational requirements instead depending on gene-locus specific additional factors, or that the binding orientation of transcription factors may generally not be relevant, but rather the event of binding itself.

**Electronic supplementary material:**

The online version of this article (doi:10.1186/s12864-016-2549-x) contains supplementary material, which is available to authorized users.

## Background

To large degree, the expression of genes is regulated at the level of transcription initiation mediated by the specific binding of protein transcription factors (TFs) to short DNA sequence motifs located in gene promoter regions, the DNA-sequence region upstream of genes. Employing both experimental [1–5] as well as bioinformatic [6–10] methods, hundreds of cis-regulatory motif sequences, partly also along with the identification of the associated transcription factors binding to them, have been determined across all model organisms and associated database resources have been created [11]. Intensive research activities have been devoted towards understanding the principles governing the specific recognition of DNA-motifs by protein transcription factors [12], their positional preferences relative to transcription start sites [13–16], their mode of action - whether to act as single entities or in combinations of different TFs and associated motifs [17, 18], as well as their evolution [19]. In turn, the principles gleaned from these studies have been applied to identify additional motifs. For example, evolutionary motif conservation proved to be a powerful approach to uncover novel motifs [20–24].

**Fig. 1:**
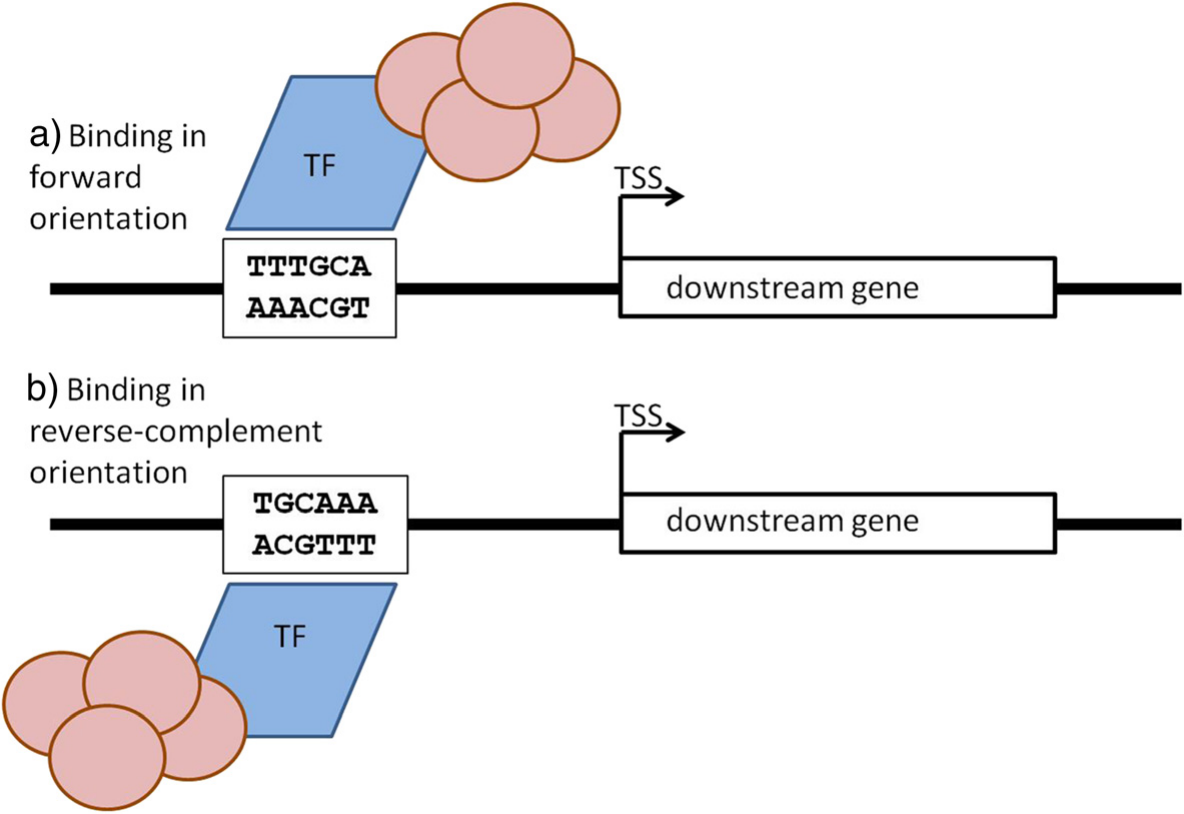
Schematic illustration of the consequences of the orientation reversal of an hypothetical, non-palindromic cis-regulatory transcription factor binding site motif. As the motif sequence is reversed in sequence direction on the opposing strand creating a reverse-complemented version of the original motif, an identical DNA interaction surface is created, but the associated transcription factor (TF) is required to bind in a 180° rotated orientation. As the TF may be asymmetric and possibly additional protein factors (denoted by light-red circles) associate non-symmetrically, the binding orientation relative to the transcription start site (TSS) of the downstream gene is reversed

Despite the large body of research on transcription factor binding sites (TFBSs), a seemingly simple question appears surprisingly unexplored, and consequently, undecided: Does the orientation of TFBSs matter? Is it important for the binding and subsequent transcriptional induction or repression of the downstream gene, whether the TFBS is present in a particular orientation such that forward and reverse-complement orientations are distinguishable and have different consequences for the transcription initiation of the downstream gene? Even though, in both orientations - forward or reverse-complement - the exact same DNA molecular interaction surface is presented to a transcription factor protein, the orientation of binding relative to the transcription start site (TSS) of the downstream gene is altered by a 180° rotation (see Fig. 1 for a schematic illustration). Transcription factors themselves may not generally possess an equivalent rotational symmetry and possibly additional protein factors may bind asymmetrically to TFs ultimately leading to the creation of the transcription initiation complex. Therefore, the inversion of the recognition motif may lead to substantial alterations of the relative positioning of interaction surfaces and, thus, the consequences on transcription initiation may perhaps be dramatic: conducive in one orientation, silent in the other.

When consulting the literature, no conclusive picture on the relevance of TFBS orientation emerges. For the TATA-box motif, a frequently occurring core-promoter element, which is typically located very close to the transcription start site (within 50 bp from the TSS), the relevance of its orientation has been addressed explicitly. The binding of the TATA-box binding protein (TBP) to its target motif is followed by the assembly of the multiprotein preinitiation complex (PIC). Thus, the TATA-box motif appears to be a prime candidate for the relevance of precise geometric orientations and spatial arrangements to become apparent. Indeed, strong orientation effects have been reported. In Drosophila, the consensus forward motif (“TATAAAAA”) was found to be associated with RNA-polymerase RNAP II transcription, while the reverse-complement motif (“TTTTTATA”) triggered RNAP III transcription, and furthermore, led to the transcription of the upstream, rather than the downstream gene [25]. Thus, motif reversal caused a transcriptional reversal as well, just as might be naively expected. However, bidirectional transcription of the forward TATA-box motif as well as forward transcription from a reverse TATA-box has also been reported [26]. Furthermore, the binding of the TATA-box binding protein (TBP) was observed to occur without orientational preference in vivo [27]. Thus, even in the case of the TATA-box core promoter motif, no definitive orientational effect has been reported. Much less so for other, general TFBS-motifs further upstream. Studies on the subject have focused on individual motifs and presented evidence for either the significance or indifference with regard to orientation. For example, orientation and spacing effects have been reported for binding sites of selected nuclear hormone receptors [28]. Similarly, viral promoters were shown to be orientation-dependent [29]. By contrast, the insulin responsive element was observed to be functional irrespective of orientation in human [30].

Experimental approaches used to determine the specifics of TF binding either do not provide sufficient sequence resolution to directly detect single TFs bound to their short recognition motif (e.g., Chip-Seq [31], nuclease-footprint [5] technologies yield sequence information on several hundreds of nucleotides and need to employ enrichment strategies to identify short motifs) or measure binding affinities to short oligonucleotides in the absence of genomic context (e.g., SELEX [1]). Novel experimental protocols and technologies are currently being introduced that yield nucleotide resolution of protein footprints in vivo allowing motif identification at much increased resolution (e.g., Chip-exo [32], Chip-nexus [33], or X-Chip-seq [34]).

Results of a study on evolutionary conservation in yeast species reporting that motif orientation relative to the orientation of its downstream genes is strongly conserved [35] may suggest that motif orientation for a given motif-gene pair matters. Though, it might be argued that preservation of a particular orientation in evolution does not necessarily mean that the alternative, reverse-complement orientation is defunct. Local, sequence-confined inversions or emergence of reverse-complement versions of a motif may simply be rare events. And once an orientation was “chosen”, evolution “kept” it. Furthermore, the question whether a particular motif exhibits orientation preferences across all its instances remains untouched by the observation that single motif instances preserve their orientation in evolution.

For regulatory motifs other than TFBSs in gene promoters such as enhancers or cis-elements in intronic regions, both orientation dependent [36] as well as orientation independent [37] activities have been reported.

Matching the ambiguity of the reported experimental findings, bioinformatics methods developed for the detection of motifs frequently do not differentiate between forward and reverse-complement orientation [38], while others do [39].

The question of orientation effects can be extended to motif pairs as TFBSs have been observed to act in combination [18]. A recent study in human revealed pair-co-operativity to be influenced by the target DNA as the recognition motifs determined for single TFs differed markedly from their respective recognition motifs when acting in combination with other transcription factors. Approximately half of all co-operative TF-pairs were found to tolerate variable spacings and/or orientations [17].

The issue of directionality has also been examined at the level of whole promoters, more specifically, core-promoters; i.e., the sequence region interval (~ +/−50 bp) around the TSS where the assembly of the transcription initiation complex is occurring. While several studies reported that (human) promoters generally act bi-directionally [40–42], a recent study challenged this view and concluded that they operate uni-directionally [43, 44].

Here, we set out to investigate the significance of motif orientation for the regulation of the expression of downstream genes pursuing a statistical approach and using *Arabidopsis thaliana* as the chosen model organism. We probed for orientation preferences and effects of reported TFBS-motifs and their pairwise combinations based on mapping statistics and, more importantly, on detected evidence of functional relevance as judged by expression effects with the rationale that all genes that harbor a given and functionally active motif should show an increased co-expression regulation compared to genes devoid of this motif in their promoter sequence. For *Arabidopsis thaliana*, all required data resources for the pursuit of our goals are well established. The genome has been sequenced and thoroughly curated and annotated [45], reliable gene models have been built permitting the identification of promoter regions, many TFBS-motifs have been reported, and thousands of gene expression profiling samples all based on the same expression detection platform are available.

While our study revealed clear evidence for the functional effect of the presence of reported TFBS-motifs on co-expression regulation of downstream genes, no significant difference was apparent with regard to motif orientation. Even though sequence regions closer to the TSS were found with increased percentages of motifs showing orientation preference, no corresponding effect on gene co-expression regulation was evident when probing genes harboring the preferred orientation for increased co-expression relative to genes with the respective motif present in its reverse-complement orientation. Furthermore, mapping preferences did not differ from random expectation and appear caused by an underlying sequence asymmetry close to the TSS revealed here based on dinucleotide frequencies. Similarly for motif pairs, motif orientation and order preferences were revealed, but no associated effect on gene expression. Our results suggest that either for the motifs considered here, no genome-wide preferred orientation exists with orientational requirements instead depending on gene-locus specific factors, or that the binding orientation of TFs relative to the transcription start site of the downstream gene may generally not be relevant, but rather the event of binding itself.

## Results

To determine whether the productive recognition of transcription factor binding site (TFBS) motifs by transcription factors (TF) leading to the subsequent expression regulation of the respective downstream effects depends on motif orientation - forward or reverse-complement relative to the coding strand of the downstream gene -, and focusing first on individual motifs, we pursued a statistical approach according to the following rationale. Relevance of motif orientation should become evident by either a pronounced occurrence asymmetry of one orientation relative to the other as the functional orientation can be expected to be conserved in evolution and therefore enriched relative to the alternative orientation not under conservation pressure. Or by an increased co-expression regulation of genes with the functional orientation of a given motif present in their promoter regions compared to gene sets containing the motif in the alternative, non-functional orientation. As it was shown that TFBSs frequently exhibit location preferences with regard to sequence distance from the transcription start site (TSS) [13–16, 35], we also compared mapping locations of both possible orientations in search of orientation dependent differences (random locations vs. preferred intervals as judged by position entropy, PE) that may further support the statistical evidence derived from mapping counts and observed co-expression.

We conducted our statistical survey using data available for the well characterized model plant *Arabidopsis thaliana.* We gathered a set of 293 non-palindromic cis-regulatory TFBS motifs with 117 of them obtained from literature/database-reported motifs (source A) and an additional set of 176 motifs originating from the Cis-BP dataset containing motifs detected experimentally in Protein Binding Assays (source B, see Methods and Additional file 1). In addition, we examined 10 core promoter motifs found enriched in the upstream regions close (50 bp) to gene TSSs in Arabidopsis ([46], source C). Promoter regions - the upstream regions of genes harboring cis-regulatory elements - were considered up to a length of 500 bp upstream of the TSSs of all nuclear-encoded genes. Based on suppressed polymorphism frequencies across many Arabidopsis accessions in this interval and given the gene density in Arabidopsis, 500 bp was shown to be a reasonable estimate of promoter length in Arabidopsis [20]. To also test regions closer to the TSS, possibly revealing stronger orientational effects as geometrical constraints on the assembly of all proteins involved in transcription initiation may become more restrictive, intervals of 250 bp and 100 bp were tested as well. With regard to mapping statistics, an even finer positional resolution of 100 bp non-overlapping sequence intervals was employed in addition. Core promoter motifs were mapped to 50 bp upstream intervals only. To set apart the core promoter region from regions of general TF-binding, the upstream interval 500-51 bp was examined for the larger motif set separately. Gene expression information was available based on hybridizations across more than 5000 samples/conditions all using the same expression profiling platform (ATH1 Affymetrix gene chip) allowing us to probe for differences in co-expression regulation depending on the presence or absence of a motif for the 20,922 genes with unique array-probe to gene mappings present on the chip.

As frequently the case in both prokaryotic and eukaryotic genomes, and shown specifically also for the genome of *Arabidopsis thaliana* [47], base compositions of both strands - the coding and the lagging strand - of the upstream region of Arabidopsis genes can be expected to be asymmetric. In particular near the TSS of Arabidopsis genes, cytosine (C) was observed to be more frequent than guanine (G) (the so-called “CG-skew”, see Additional file 2: Figure S1 for skew ratios plotted for the 500 bp upstream intervals of *Arabidopsis thaliana*). Thus, when assessing actual orientation-dependent mapping statistics, any mapping count asymmetries have to account for this orientation-dependent base-frequency difference. We employed a rigorous binomial testing with expected random mapping orientation ratios set according to the actual base compositions of the respective sequence region interval under investigation (Table 1, see Methods, Eq. 1).

**Table 1:** Base compositions

Upstream sequence interval [nt] / composition [%]	A	C	G	T
−500 to −1	33.9	16.8	15.8	33.4
−250 to −1	33.9	17.4	15.5	33.1
−500 to −401	33.6	16.3	16.5	33.6
−400 to −301	34.0	16.0	16.0	34.0
−300 to −201	34.3	16.1	15.6	34.0
−200 to −101	34.4	16.8	15.7	33.1
−100 to −1	33.3	18.7	15.4	32.5
−50 to −1	33.0	19.4	15.1	32.5
−500 to −51	34.0	16.5	15.9	33.6
TFBS motifs	29.4	24.9	20.9	24.8

Base composition of the intervals of upstream regions of Arabidopsis genes (coding strand) and of the TFBS motifs used in this study. As motifs are frequently reported in both the forward and reverse-complement orientation, the directionality reported as “forward” was chosen. Composition of the reverse-complement sequence version can be imputed from the given percentages based on canonical base-pairings A-T and G-C. In case of ambiguous bases as part of the sequence, correspondingly allowed individual bases A, C, G, or T, were counted fractionally

As many motifs were tested (293 motifs, 10 core-promoter motifs), a proper multiple testing correction needs to be applied, done typically, and performed here as well, by applying false discovery rate thresholds [48]. By compiling the same mapping and expression statistics for random motif versions following the same motif length distribution, we added an additional layer of control. As background base compositions for the creation of random motifs, we took both the base compositions as observed in the set of actual motifs as well as compositions computed from the respective gene-upstream region intervals under investigation. As listed in Table 1, motif and general upstream reference base compositions differ substantially with TFBSs exhibiting increased C and G frequencies with correspondingly lowered proportions of A and T. Furthermore, a pronounced enrichment of C in upstream regions closer to the TSS is noticeable (see also Additional file 2: Figure S1). Thus, testing both background distributions separately appears indicated.

### Marked effects of motif presence vs. absence on the co-regulation of downstream genes

**Fig. 2:**
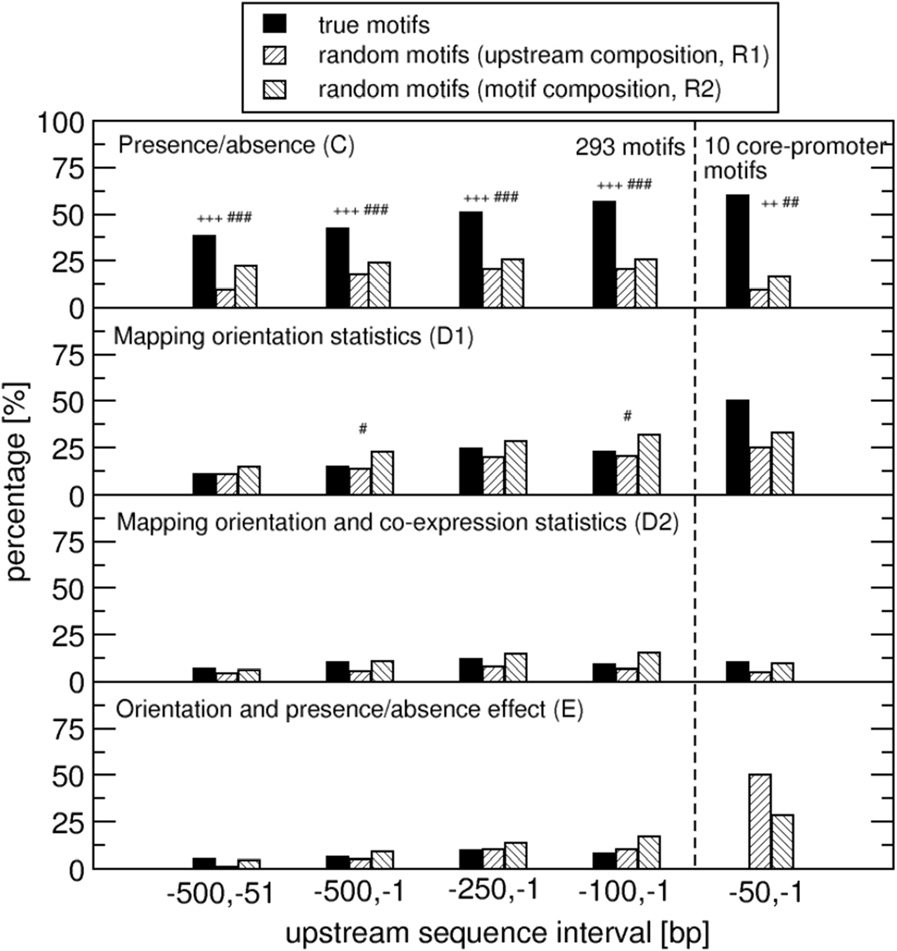
Visualization of the results statistics reported in Table 2 and (Additional file 2: Table S1). Percentages of motifs passing through the various filter criteria (C, D1, D2, and E) as explained in table legend 2 are plotted for the different upstream sequence intervals considered in this study and true motif sets (293 upstream elements, 10 core promoter elements). Results statistics are compared to sets of randomized motifs generated by either assuming base composition as observed in the respective upstream sequence interval (R1) or as observed in true motifs (R2) (see Methods, and Additional file 2: Table S1). Significant differences are annotated as “+”/”#” if percentages obtained for true motifs differed from R1/R2-randomized sets, respectively, with triple-symbols indicating *p* < 0.001, double-symbols *p* < 0.01, and single-symbols *p* < 0.05 after correcting for multiple testing

We first checked whether the cis-regulatory effect of the individual motifs in our set can be detected by the implemented co-expression test regardless of motif orientation. For all genes whose promoter sequence contain a given motif regardless of orientation, we computed all pairwise Pearson correlation coefficients between the respective normalized expression values across all 5295 gene-chip hybridizations representing a large collection of different experimental conditions applied to *Arabidopsis thaliana*. Because the presence of a given motif should cause the respective downstream genes to be co-regulated under certain conditions, the resulting distribution of correlation coefficients should be shifted to larger positive values compared to correlation coefficients obtained for gene pairs not containing the motif at all, neither in forward nor reverse-complement orientation. (All results reported in this paragraph are listed in Table 2 and displayed in Fig. 2.) Indeed, 42.4 % (*N* = 112) of all tested motifs mapping to the 500 bp gene-upstream regions resulted in significant correlation differences between the motif-present and motif-absent set (Table 2, Fig. 2). For significance criteria, please refer to the legend of Table 2 and the Method section. When repeating the analysis for randomly generated motifs, significantly fewer motifs were tested positive in this expression assay. Assuming base compositions according to the average composition of the 500 nt upstream coding strand, only 17.7 % (F_U_) revealed an effect above the implemented thresholds, significantly less than for the set of true motifs (p_U_ = 3.1E-16). Taking random motifs with the same base compositions as actual motifs, only 23.9 % (F_M_) of the motifs tested positive (p_M_ = 7.3E-09 for the fractional difference compared to the 42.4 % for actual motifs based on Fisher’s exact test). The percentage of positively tested motifs further increased when confining the considered upstream regions to segments closer to the transcription start site (TSS) with 51.1 % (F_U_ = 20.7 %, p_U_ = 4.4E-21; F_M_ = 25.5 %, p_M_ = 9.7E-16) and 56.7 % (F_U_ = 20.7 %, p_U_ = 7.7E-17; F_M_ = 26.0 %, p_M_ = 6.4E-19) positively confirmed motifs when considering 250 bp and 100 bp upstream regions, respectively (Table 2, Fig. 2). This percentage was lower, but still significantly higher than expected by chance (38.4 %, F_U_ = 15.2 %, p_U_ = 1.4E-15; F_M_ = 22.2 %, p_M_ = 6.4E-19) for the interval that excludes the assumed core-promoter region (−500 bp, −51 bp). Thus, motifs with locations closer to the TSS appear to exert a stronger cis-regulatory influence on the downstream genes.

**Table 2:** Motif mapping and co-expression statistics

A) Up-stream sequence interval [bp]	B) Motifs	C) Presence/ absence statistic	D) Mapping orientation statistic, D1/D2/D3	E) Orientation and presence/ absence effect (% of C)	F) Set E with PE-filter	G) Motifs matching criteria E) and, indicated by (*), F)
−500, −1	264	112 (42.4 %)	39 (14.7 %)^#^/ 27 (10.2 %)/ 16 (6.1 %)	7 (6.2 % of 112)	6	M0007_1.01*, M0252_1.01*, M0576_1.01, M1180_1.01*, ABRE-like_binding_site_motif*, ACGTABREMOTIFA2OSEM*,, GADOWNAT*
−250, −1	264	135 (51.1 %)	65 (24.6 %)/ 32 (12.1 %)/ NA	13 (9.6 % of 135)	NA	M0007_1.01, M0023_1.01, M0036_1.01, M0078_1.01, M0264_1.01, M0516_1.01, M0576_1.01, M0578_1.01, M0770_1.01, M1180_1.01, ACGTABREMOTIFA2OSEM, Box_II_promoter_motif, LEAFYATAG
−100, −1	247	140 (56.7 %)	57 (23.1 %)^#^/ 23 (9.3 %)/ NA	11 (7.8 % of 140)	NA	M0014_1.01, M0016_1.01, M0019_1.01, M0031_1.01, M0078_1.01, M0081_1.01, M1180_1.01, Bellringer/replumless/ pennywise_BS1_IN_AG, Box_II_promoter motif, LTRECOREATCOR15, MYB_binding_site motif
−500, −51	260	100 (38.4 %)	28 (10.8 %)/ 18 (6.9 %) / NA	5 (5 % of 100)	NA	M0007_1.01, M0252_1.01, ACGTABREMOTIFA2OSEM, GADOWNAT, GAREAT
Core promoter motifs						
−50, −1	10	6 (60 %)	5 (50 %) / 1 (TATA-box, 10 %) / NA	0 (0 % of 4)	NA	No significant motifs according to filter criteria E detected

Motif mapping and co-expression analysis results. Table columns list information on A) the interval of the considered upstream regions, B) the number of considered motifs with valid observations (Note that the number of considered motifs (column B) is less than the reported set size of 293 motifs of initially compiled motifs (see Methods) and differs between test settings as for the motifs that dropped out either i) mapping counts were insufficient, or ii) no probes were present on the ATH1 chip for the respective downstream gene, and we report the results of motifs with complete information across all tests (mapping, expression, and, in the case of 500 bp upstream region, position entropy) only.), C) Number (percentage) of motifs with significant co-expression differences between genes containing the genes upstream regardless of direction compared to genes not containing the motif at all (neither in forward nor reverse-complement orientation) with thresholds p_r_diff_ < 0.05 and Cohen’s d > 0.01. D) Motif mapping statistics with D1 indicating the number of motifs with significant orientation preference (p_orient_ < 0.05), D2 - subset of D1 meeting also the criteria of significant co-expression differences (p_r_diff_ < 0.05) with higher intra-set correlations in the set corresponding to the preferred mapping orientation, and, in addition (D3), lowered positional entropy (PE) in the preferred orientation. As no positional entropies were computed for the shorter upstream intervals of length 250 bp and 100 bp, D3 is not provided for those sets. E) Filter criteria D2 applied only to the subset of motifs with evidence of significant presence/absence effect (column B) (Note that the multiple testing correction was adjusted accordingly.) F) Subset of E that also exhibit lowered positional entropy (PE) in the preferred orientation (Filter criteria D3, applied to upstream regions of length 500 bp only as positional preferences lose their meaning for smaller considered sequence intervals). G) Actual motif names fulfilling filter criteria E, and if indicated by asterisks, F. Underlined values denote counts and percentages significantly different from random expectation (*p* < 0.01) based on Fisher’s exact tests with randomly expected counts determined from mapping statistics and expression analyses obtained for sets of random motifs with compositions based on the considered upstream regional interval as well as upstream motifs (Additional file 2: Table S1; see Methods); i.e., found significant relative to both randomized sets R1 and R2. § - significantly different relative to upstream-composition-based randomization (R1 Additional file 2: Table S1) (This case was not observed), # - significantly different relative to motif-composition-based randomization (R2 Additional file 2: Table S1) with significance judged after correcting for multiple testing

Based on the results of the initial presence/absence test, we conclude that the implemented co-expression screen is sufficiently sensitive to detect cis-regulatory effects and that the set of used motifs is indeed acting cis-regulatorily. Evidently, in this first test, the contrasted sets differed more significantly with respect to the assignment of motif presence or absence compared to the subsequent tests, in which we compared gene sets that contained a given motif in one orientation only relative to the set of genes with motif mappings in the alternative, reverse-complement orientation only. Thus, the motif is present in both situations, but its orientation is reversed and complemented in one case. As argued above, this creates the same interaction surface for the cognate transcription factor, but alters its orientation by a 180° rotation relative to the location of the TSS of the downstream gene (Fig. 1).

First, we compiled the associated orientation-specific motif mapping statistics. Regardless of actual effects on cis-regulatory gene expression regulation, any statistically significant asymmetries with regard to mapping frequencies would be indicative of the relevance of motif orientation. (All results described in the subsequent paragraphs are presented in detail in Table 2 and in (Additional file 1: Table S1), and are displayed graphically in Fig. 2). 

### Mapping statistics reveal no motif orientation effects

Based on orientation-specific mapping statistics and irrespective of any evidence of gene expression regulatory effects and first focusing on the 500 bp-upstream region, 14.7 % (*N* = 39) of all motifs were found to exhibit a pronounced orientation-specific mapping bias (see Methods), i.e., to occur in either the forward or reverse-complement orientation more often than randomly expected. No significant difference was detected when comparing this percentage to random motifs based on upstream-sequence composition (13.7 %; Fisher exact test, p_U_ = 0.86). The percentage of random motifs assuming actual motif compositions as background was even slightly increased, albeit at marginal significance levels only (22.7 %; Fisher exact test, p_M_ = 0.015).

### No evidence of orientation dependence on cis-regulatory gene expression regulation

Next, we combined the mapping filter with possible evidence of cis-regulatory gene expression regulation, i.e., motifs with a preferred mapping orientation also reveal a marked increased co-expression amongst the genes harboring the motif in this orientation. Again, no statistically significant differences were determined when testing the actual motif set and comparing it to the random motif sets generated using the two different background base compositions. Of all tested motifs, 10.2 % of actual motifs were identified to pass both the mapping and co-expression filter criteria, but no significant difference relative to random motifs was detected (p_U_ = 0.06 (5.7 %), p_M_ = 1 (11.9 %)).

We furthermore applied an additional filter by imposing evidence of preferred motif location in the orientation that was detected preferred based on the mapping statistics and by a positive co-expression result. Location preferences were judged by the introduced positional entropy (PE, Eq. 2, see Methods), which should be smaller for motifs confined to particular positional intervals relative to motifs with random motif locations in the upstream region. Again, actual motifs did not pass this third filter at significantly higher rates (6.1 %) than the two random motif control sets (p_U_ = 0.13 (3.8 %), p_M_ = 0.59 (7.3 %)).

We then confined the set of considered motifs for the detection of orientation and location effects to only those 112 motifs that were tested positive for a significant cis-regulatory effect on their downstream genes (column C of Table 2) yielding seven (6.2 %) motifs and six, when combined with the position-entropy filter (Table 2). No significant differences were found with regard to relative motif counts passing the correlation or, in addition, the positional entropy filter compared to the two random motif sets.

We interrogated motif mapping statistics considering shorter upstream segments of length 250 bp and 100 bp, as well as the upstream region (−500 bp,−51 bp); i.e., the interval without the immediate core-promoter region. Significantly more motifs showed orientation preference (24.6 %, Fisher exact test, *p* = 0.006 and 23.1 %, Fisher exact test, *p* = 0.017, in the 250 bp/100 bp interval, respectively, compared to 14.7 % in the 500 bp interval). However, this increase did not translate into associated effects when filtered for expression and not difference with regard to random motif statistics was evident. Again, similar percentages of actual motifs were observed to pass the imposed filterers compared to random motif versions (Table 2, Fig. 2, Additional file 2: Table S1). In summary, contrary to expectation, no increasing relevance of motif orientation was evident when considering upstream intervals closer to the TSS. For the upstream interval (−500 bp,−51 bp), the percentage of motifs passing the series of filters was lower than for those regions that include the 50 bp immediately upstream of the TSS (Table 2) underlining the importance of the core-promoter region for TF binding or motif recognition in general.

Despite the absence of any significant orientation or position effects associated with the set of actual motifs as a whole, we provide the motif identifiers for the motifs that passed the most stringent filter criteria in Table 2 (column G). For example, the motif M1180_1.01 (consensus sequence: “KGGTTAAM”) was detected across all imposed filter criteria and in all but one (−500 bp,−51 bp) considered upstream region intervals.

Summarizing the results obtained for the mapping statistics and performed co-expression analyses for the set of 293 cis-regulatory motifs, we note that motif presence vs. absence has a pronounced effect on co-expression of the corresponding gene sets and across all upstream regional intervals (Fig. 2, filter/panel C). By contrast, no significant (with two borderline exceptions, see below) differences were observed with regard to the percentage of true motifs passing the various filters implemented to test for relevance of motif orientation (Fig. 2, filters/panels D1, D2, E). However, while not significant at the individual test level, it can be noted that, as a trend, random motifs based on upstream sequence compositions passed the filters at slightly lower percentages than true motifs, while random motifs constructed using actual motif compositions as background passed these filters at even slightly increased percentages compared to true motifs (Fig. 2, Additional file 2: Table S1). This includes the two borderline significant differences (Fig. 2, filter/panel D1). True motifs display slightly higher tendencies to be orientation-sensitive than expected when assuming background base compositions derived from general sequence compositions in the respective upstream sequence intervals, but slightly less than expected based on motif compositions themselves. Thus, true motifs are even less orientation-sensitive than what is possible based on composition alone. Their actual sequences render them slightly less orientation-sensitive than what is randomly expected.

For all motifs, comprehensive result tables are provided as Additional file 1.

### Positional dependence of motif orientation preferences in conjunction with underlying sequence directionalities

We observed a significant increase of the percentage of motifs exhibiting orientation preferences when confining the analysis to sequence regions of length 250 nt (24.6 %) and 100 nt (23.1 %) compared to 500 nt (14.7 %, Table 2 and Fig. 2, metric D1) upstream of the TSS. This elevated orientational preference seems to suggest an increased relevance of motif orientation at positions closer to the TSS, even though we did not detect this increase to be associated with a significant effect with regard to gene co-expression regulation, which was in line with random expectation. To further explore the mapping statistics and to reconcile this apparent contradiction, we analyzed the motif mapping statistics at higher positional resolution employing non-overlapping sequence intervals of length 100 nt across the entire considered upstream promoter region (500 nt).

**Fig. 3:**
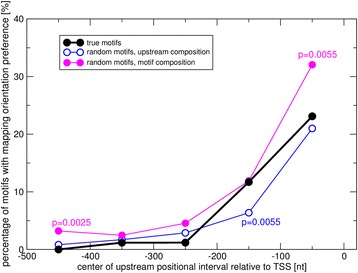
Motif orientation preferences as a function of distance from the transcription start site (TSS). Based on mapping statistics alone, the percentage of motifs with significant (binomial test *p* < 0.05, Benjamini-Hochberg (BH) corrected) to upstream intervals of length 100 nt at different distances from the transcription start site (TSS). Statics were generated for the set of actual motifs (*black line*) as well as randomized motifs based on the composition of the respective interval (*blue line*) or based on motif-base compositions (*magenta line*). For randomized motifs, 10 repeats were performed and the average percentage of motifs with orientation preference computed. *P*-values indicate significant departure of the respective random expectation from true motifs (binomial test, BH-corrected). Motifs were required to map ten times or more to the respective upstream regional interval or ignored otherwise

Again, the set of true motifs was found to exhibit pronounced increased orientational preferences at sequence positions closer to the TSS (Fig. 3, black curve). However, this trend was paralleled by an equally strong increase of orientational preferences of randomized motif versions. Assuming as sources of random motif generation base compositions as observed either in the respective sequence intervals or actual motifs, both resulted in equally pronounced increases of the percentage of motifs showing orientational preferences that are – except for the highlighted cases in Fig. 3 – not significantly different than the percentages observed for true motifs. On average, random motifs based on motif-compositions were found with even higher rates of orientation preferences than actual motifs (Fig. 3, magenta curve), while background-sequence-composition-based random motifs showed reduced orientation bias in intervals closer to the TSS, yet reaching significance in the interval (−200…−101 nt) only, and elevated, albeit insignificantly, percentages in regions further upstream (Fig. 3, blue curve).

**Fig. 4:**
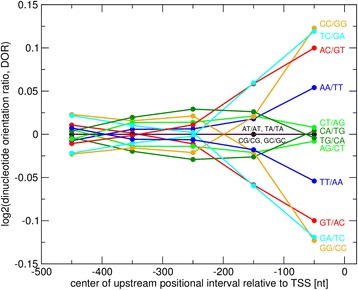
Dinucleotide orientational asymmetries in gene upstream regions. For five upstream regional intervals of length 100 nt (−500..-401, −400..-301,…,-100..-1), logarithmic (base 2) dinucleotide orientation ratios (DORs, see Methods, Eq. 4) of the observed-vs-expected frequency ratios of all possible dinucleotides of its forward relative to the respective reverse-complement version are plotted. Observed-vs-expected frequency ratios measure the departure of actually observed dinucleotide frequencies versus their estimated frequencies based on single base frequencies alone; i.e., treating them as independent events (see Methods for details). Thus, deviations from zero indicate evidence of conditional probability differences between the forward and reverse-complement dinucleotide version and are indicative of orientational preferences. For palindromic dinucleotides (AT,CG,GC,TA), this log-ratio computes as zero. Ratios are plotted for dinucleotide combinations with pairs constituting respective inverse ratios (e.g., TC/GA and GA/TC) necessarily resulting in symmetric graphs

At first, the trend towards increased evidence of orientation preference even for randomized motifs is surprising. As we have implemented measures to correct for simple compositional effects – expected ratios of forward to reverse-complement mappings were adjusted for base compositions (Eq. 1), and one set of randomized motifs was furthermore based on background base compositions as observed in the respective sequence interval – the upward trend cannot be explained by compositional shifts. Instead, there must be higher order sequence compositional biases resulting in an underlying orientational asymmetry of sequence regions close to the TSS. Therefore, we analyzed dinucleotide frequencies as the next level of positional dependence of base frequencies. Single base composition alone can be considered zero-order (no dependence of base frequencies on the identity of neighboring bases), while dinucleotides capture first-order effects asking whether the identity of a base at a given first position influences the identity of the next consecutive, second base. Indeed, when inspecting upstream regions for dinucleotide frequencies corrected for zero-order compositional bias (see Methods, Eqs. 3 and 4), we detected pronounced orientational preferences of dinucleotides at sequence intervals closer than 200 nt, and even more strongly, in the first 100 nt upstream positions relative to the TSS (Fig. 4). For a number of dinucleotides, their respective forward version occurs at different frequencies than the corresponding reverse-complement dinucleotide (indicated by departures of the curves from the zero line in Fig. 4) with this imbalance not present at sequence intervals further upstream (curves cluster around zero in Fig. 4). For example, the dinucleotide “TC” is found 8.7 % more often in the 100 nt upstream of genes than its reverse-complement “GA”. It is more likely for a “C” to occur when preceded by a “T”, than it is for an “A” to follow a “G”, the succession of bases of the corresponding reverse-complement dinucleotide. We wish to emphasize again that this effect is composition corrected (Eq. 3). Similar directionalities were observed for a number of additional dinucleotides (CC/GG, AC/GT, AA/TT, Fig. 4), Thus, the upstream regions close to the TSS exhibit intrinsic orientation biases causing zero-order random motifs to map accordingly resulting in the observed increased orientation preference (Fig. 3).

### Core-promoter motifs, TATA-box motif

As remarked in the introduction, because of its proximity to the TSS and likely stringent packing constraints of the assembly of the transcription initiation complex, core promoter motifs, and the TATA-box motif in particular, represent prime candidate motifs for any orientation effects to become apparent. When inspecting the mapping statistic determined for ten reported Arabidopsis core promoter motifs [46] to the 50 bp upstream sequence regions, for five motifs (“ARARAVAAAR”, “TCDTCDTC”, “AAACCCTARH”, “ARGCCCAW”, “TATAAA”) pronounced orientation preferences were detected (50 % compared to 25 %, p_U_ = 0.57, and 33 %, p_M_ = 0.47, expected randomly assuming the two types of random control sets), but for only one motif (TATA-box motif, “TATAAA”), a consistent expression co-regulation was determined (Table 2). For the TATA-box motif, in particular, there is a very significant preference for the canonical forward motif definition compared to its reverse-complemented version (*p* = 1.1E-129). Furthermore, genes with the forward (fw) TATA-box have increased correlation levels between them compared to the set of genes with the reverse-complement (rc) orientation (average r_fw_ = 0.008 vs. average r_rc_ = 0.004, *p* = 2.35E-21, Cohen’s *d* = 0.023). While significant, the difference in magnitude (Cohen’s d) is small. The TATA-box motif was reported to be associated specifically with stress response genes (at least in yeast, [49]), and thus expecting some level of co-expression between them appears reasonable. However, the TATA-box is still a rather non-specific motif acting as a core-promoter element − 5153 (15.5 %) of all Arabidopsis genes contain at least one TATA-box motif in their 50 bp upstream region, with TATA-box defined as “TATAAA” and its reverse-complement “TTTATA” - and as very many conditions are compared (>5000 hybridization samples), large correlation differences cannot be expected. The TATA-box motif also exhibits increased location preferences when tested in 500 bp upstream intervals in the forward compared to the reverse-complemented version with corresponding positional entropies PE_fw_ = 2.29, PE_rc_ = 2.31. Again, the difference is small, but consistent with expectation as smaller values of PE indicate a confinement to preferred positional intervals indicative of position-specific effects. However, and as previously noted as a possible reflection of the “general-purpose” and core-promoter functionality of the TATA-box motif [20], when comparing the co-expression of genes containing the TATA-box motif irrespective of orientation in their 50 bp upstream sequence to genes not containing the element at all in this sequence interval, co-expression was even increased in the latter set, contradicting expectation (average r_present_ = 0.006 vs. average r_absent_ = 0.012, *p* = 4.3E-44, Cohen’s *d* = −0.03). Thus, the conclusions remain partially ambiguous. Orientation effects of the TATA-box motif appear present, yet its relevance for coordinating gene expression by way of presence/absence does not seem to be consistently supported given the data and analyses presented here.

Inspecting all core-promoter motifs across all imposed filters, a pronounced presence/absence affect was evident as observed for the general set of motifs (Fig. 2, filter/panel C). While preference for a particular orientation was observed (Fig. 2, filter/panel D1), significance could not be established. Furthermore, generally no additional evidence for orientation-sensitivity was evident (Fig. 2, filters/panels D2, E). (Detail result statistics on core-promoter motifs are presented as (Additional files 1 and 2)).

### Motif pairs

Cis-regulatory motifs were reported to frequently operate in combination [18, 50]. Hence, we investigated whether orientation effects become apparent when considering motif pairs. For this analysis, we further reduced the motif set to only those 62 motifs (not considering the core-promoter motifs) that are truly not contained in any other (longer) motif even when considering all possible sequence variants associated with ambiguous bases as part of the motif definitions (see Methods). Otherwise, two motifs would be found unduly coupled (found in the same promoter) as the same mapping positions are (possibly) identified. Furthermore, deciding which of the two respective transcription factors binds to this region may be ambiguous. At the same time, this lessened the penalty associated with the multiple testing correction as the number of possible pairs scales quadratically with the number of motifs. We first probed all detected motif combinations found in the upstream regions of the same gene for statistical enrichment (gene set overlap) and then examined all eight possible relative orientations of two motifs with respect to their sequence order (position in the upstream region) and orientation (forward or reverse-complement). Here, we considered all motif mappings to the upstream interval of −500 bp to −51 bp to exclude the core promoter region (~50 bp upstream of the TSS), which harbors its own characteristic set of motifs with pronounced location preferences such that they would always be found downstream of another motif. Non-overlapping motifs (mapping position) only were considered to constitute a valid candidate motif pair.

Testing all 1596 possible motif pairs (including same-motif pairs) associated with the 56 motifs found to map to the considered upstream sequence regions, yielded 13 motif pairs (0.8 % of all 1596) comprising 14 unique motifs found to co-occur in the same upstream region significantly more often than expected by chance and thus may operate in combination (Table 3). Two pairs were same-motif pairs: TELO-box promoter motif (“AAACCCTAA”) and Cis-BP motif M2220_1.01 (“HCACGCGCT”). Two motifs stand out, the Bellringer/replumless/pennywise BS1 IN AG motif (“AAATTAAA”) and the Cis-BP motif M0758_1.01 (“HMWTWAATGH”) found in four motif pairs each. While co-occurring more frequently than expected, only two of the 13 motif pairs resulted in noticeable co-expression difference when comparing the gene sets harboring both motifs versus the set of genes with only one of them (Table 3). For the same-motif pair TELO-box promoter motif, genes harboring the motif repeatedly in their upstream region are significantly more co-expressed (*p* = 3.03E-42) and with a pronounced effect size (Cohen’s = 2.82E-01 = 28.2 %) than genes with only one motif instance. Genes with the EveningElement promoter motif in combination with the Bellringer/replumless/pennywise_BS1_IN_AG motif also showed evidence of gene co-expression regulation (*p* = 2.21E-03), albeit the effect size was small (Cohen’s *d* = 2.51E-02 = 2.51 %).

**Table 3:** Motif pair co-occurrence statistics

Motif 1	Motif 2	Genes with motif 1	Genes with motif 2	Genes with both motifs	*p*-value (BH corrected)	p_BH_ Expr	Cohen’s d
M0758_1.01	Bellringer/replumless/pennywise_BS1_IN_AG	6845	6630	1619	0.00E + 00	1.00E + 00	6.51E-03
TELO-box_promoter motif	TELO-box_promoter motif	1472	1472	146	0.00E + 00	3.03E-42	2.82E-01
SBOXATRBCS	M0769_1.01	276	2106	45	2.61E-06	1.00E + 00	−1.58E-02
M2220_1.01	M2220_1.01	213	213	12	5.86E-06	1.00E + 00	1.04E-01
M0758_1.01	ATHB6_binding_site motif	6845	666	187	3.92E-04	1.31E-01	−1.13E-02
Bellringer/replumless/pennywise_BS1_IN_AG	M2241_1.01	6630	2342	555	3.92-04	1.00E + 00	2.01E-03
Bellringer/replumless/pennywise_BS1_IN_AG	ATHB6_binding_site motif	6630	666	180	8.79E-04	1.00E + 00	−1.56E-02
MYB1_binding_site motif	SBOXATRBCS	912	276	22	1.55E-03	1.00E + 00	−3.43E-02
M0758_1.01	SORLREP3	6845	1520	376	4.49E-03	5.17E-01	7.62E-03
EveningElement_promoter motif	Bellringer/replumless/pennywise_BS1_IN_AG	1249	6630	305	5.61E-03	2.21E-03	2.51E-02
ATHB6_binding_site motif	M2241_1.01	666	2342	74	8.86E-03	1.00E + 00	1.82E-02
M0758_1.01	M2241_1.01	6845	2342	552	1.42E-02	1.74E-01	−6.70E-03
TELO-box promoter motif	TL1ATSAR	1472	85	12	4.01E-02	1.00E + 00	3.22E-01

Motif pairs found to co-occur more often than expected in the same upstream regions (p_BH, hypergeometric_ <0.05, where BH denotes correction for multiple testing based on Benjamini-Hochberg [48]). Co-occurrences were counted only if motifs were found not to overlap with regard to their mapping position. P_BH_Expr is the *p*-value of the detected co-expression difference of genes containing both motifs compared to those containing only one of them in their upstream region with Cohen’s d referring to the associated effect size. Motif pairs with more than five co-occurrences are reported only. In case of same-pair motifs, genes with the respective motif found repeatedly in their upstream sequence were compared to genes harboring the motif only once

With regard to relevance of orientation when considering motif pairs, we first tested for motif order. Are there motif pairs for which their positional order in the upstream sequences shows preferences, and if so, does this correspond to differences of regulatory effects as judged by increased co-expression of genes with the motif pair positioned in the preferred order? A total of 624 distinct motif pairs of which 27 are same-motif pairs were detected with promoters in which both are present simultaneously. Of those, and requiring 50 or more instances (to ensure statistical robustness and to lessen the multiple testing penalty), 12 pairs (2.0 % of 597 all pairs consisting of two different motifs) were found to exhibit pronounced motif order preferences (Table 4), with only one pair (SORLREP3, M0758_1.01) having been identified already as enriched in upstream regions (Table 3). However, this did not translate into corresponding co-expression effects. For a single motif pair only, significance, albeit low, is achieved (TELO-box promoter motif/ Bellringer/replumless/pennywise BS1_IN_AG) and co-expression is increased for the motif order observed to be preferred (8 of the 12 pairs show consistent effect direction (*p* = 0.39). Thus, while motif pairs with pronounced order preferences were detected, their relevance as judged by co-expression could generally not be established.

**Table 4:** Motif pairs with pronounced motif order preferences

Motif 1	Motif 2	Motif 1 - Motif 2				Motif 2 -Motif 1				p_BH_ Binom	p_BH_ Expr	Cohen’s d
		++	+−	--	−+	++	+−	--	−+			
M2217_1.01	M0758_1.01	53	60	49	39	99	65	67	77	2.44E-04	4.32E-01	−1.81E-02
M2217_1.01	M2241_1.01	17	12	16	6	32	23	18	36	2.66E-04	4.32E-01	−5.83E-02
Gap-box motif	TELO-box_promoter motif	30	10	43	11	8	12	18	5	5.28E-04	3.78E-01	−6.42E-02
Bellringer/replumless/pennywise_BS1_IN_AG	M2241_1.01	89	70	79	78	97	88	99	134	4.77E-03	3.78E-01	−3.05E-02
M0758_1.01	SORLREP3	77	64	79	74	48	66	51	55	2.12E-02	3.78E-01	2.90E-02
M0769_1.01	MYB1_binding_site motif	22	12	8	7	7	3	4	7	2.12E-02	7.67E-01	−3.65E-02
M2241_1.01	M1224_1.01	4	4	8	5	9	12	14	13	2.24E-02	7.58E-01	1.17E-02
Bellringer/replumless/pennywise_BS1_IN_AG	M2251_1.01	35	25	22	23	28	9	13	14	2.25E-02	7.58E-01	2.62E-02
M2251_1.01	M1561_1.01	5	4	5	6	16	7	6	17	2.25E-02	7.58E-01	−4.86E-02
TELO-box_promoter motif	Bellringer/replumless/pennywise_BS1_IN_AG	32	29	32	26	40	41	44	45	3.23E-02	1.37E-02	−1.14E-01
M0758_1.01	M2251_1.01	39	36	25	22	27	25	19	9	3.30E-02	7.20E-01	8.89E-04
SORLREP3	M2241_1.01	20	13	10	17	32	23	20	22	3.30E-02	4.08E-01	3.15E-02

Motif pairs with pronounced motif order preferences. Motif pairs were considered in both relative positions (motif 1 upstream of motif 2 or the reverse). For both arrangements, four different motif combinations of motif orientations are possible with “+”/”-“denoting forward and reverse-complement direction, respectively. P_BH_Binom is the multiple-testing corrected *p*-value obtained from testing the order preference based on binomial test with *p* = 0.5 as the assumed background probability (no preferred motif order) and based on the summed up counts (over all four orientation arrangements) for the two motif orders respectively. P_BH_Expr is the *p*-value of the detected co-expression difference of genes in which the motif pair was found in one order relative to genes with the inverse motif order (genes found in both sets were excluded). Cohen’s d refers to the associated effect size with positive signs signifying increased co-expression among genes with motif order 1–2, negative signs among genes with motif order 2–1. Motif pairs with 50 or more occurrences were considered only. Note that for same-motif pairs, this analysis has no meaning, hence they were not considered

**Fig. 5:**
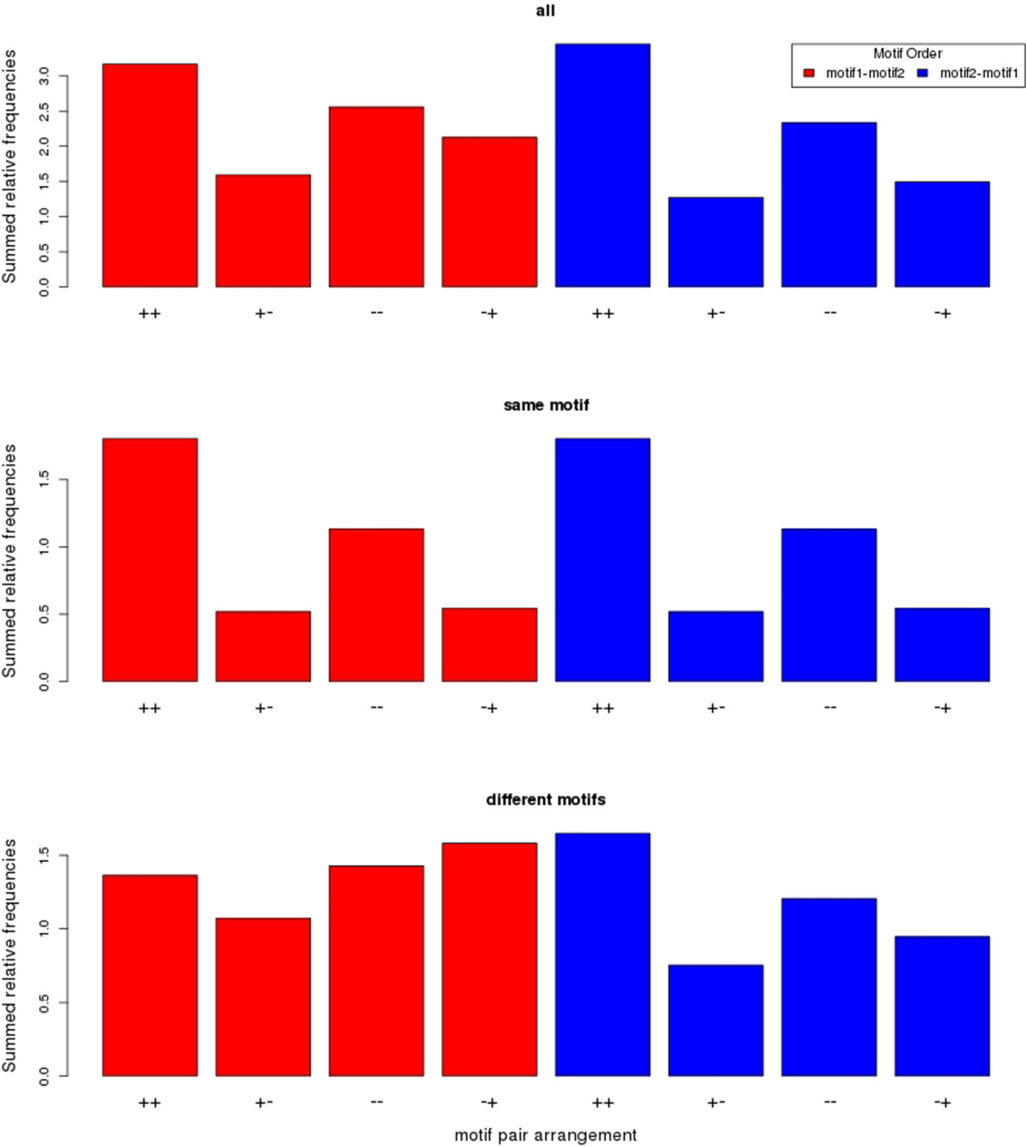
Preferred motif pair arrangements. For all 18 motif pairs with non-random arrangement distribution, relative frequencies were computed; i.e., counts per arrangement divided by the total count for all eight possible arrangements followed by a summation for each arrangement type across all 18 motifs. Plotted are the resulting summed-up relative frequencies for the two possible motif orders with regard to sequence position and orientation where “+” denotes forward, and “-“reverse-complement orientation. Preferences for co-directionally aligned orientations, i.e., both in forward or both in reverse-complement orientation are evident. The central plot entitled “same motif” shows the relative counts for same-motifs only (Note that the order does not matter and the associated frequencies are identical.), whereas the plot “different motifs” shows the data for two different motifs considered a pair

Next, we examined whether occurrences of particular orientations of two motifs when treated as a pair show significant departure from random expectation. Given two motifs, eight different arrangements are possible resulting from the combinations of motif order and motif orientation (forward/ reverse-complement) that are expected to occur at the same frequency by chance. Assessing the significance of non-randomness of occurrences of particular arrangements by a single entropy-based test (see Methods) yielded 27 motif pairs (4.3 %) with significant non-random arrangement patterns, i.e., one or several of the eight arrangements occur more often than expected by chance at the expense of others. As this may also result from order preferences alone (see above, Table 4), we further required that motif pairs also show non-random occurrence-patterns for the two possible motif orders individually resulting in 18 (2.9 %) motifs with non-random arrangement preferences of which 8 are same-motif pairs (Table 5). Noteworthy, a preference for co-directionally aligned orientations is evident when examining the summed up relative frequencies of counts per arrangement type (Fig. 5). Arrangements in which the two motifs in the pair are either both oriented in forward or both in reverse-complement direction occur frequently. This preference is particularly pronounced for same-motif pairs (Fig. 5, barplot “same motif”) and less obvious for pairs composed of two different motifs (Fig. 5, barplot “different motifs”). However, when testing whether genes harboring the most frequent arrangement of a given motif pair in their upstream region relative to those associated with the three least frequent arrangements, no pair proved motif-pair-arrangement sensitive as significance was either not established, or the effect was reverse; i.e., increased co-expression was found for the least frequent motif pair arrangements. As concluded for motif pair order, also when combined with motif orientation, pairs with significant preferences are discernable, but their influence on gene expression co-regulation cannot be established.

**Table 5:** Motif pairs with pronounced non-random motif order and orientation preferences

Motif 1	Motif 2	Motif 1 - Motif 2				Motif 2 -Motif 1				p_BH_ Entropy	p_BH_ Expr	Cohen’s d
		++	+−	--	−+	++	+−	--	−+			
Bellringer/replumless/pennywise_BS1_IN_AG	Bellringer/replumless/pennywise_BS1_IN_AG	405	283	322	316	405	283	322	316	0.00E + 00	6.99E-01	1.82E-02
TELO-box_promoter motif	TELO-box_promoter motif	200	35	165	10	200	35	165	10	0.00E + 00	2.96E-02	−1.96E-01
M2217_1.01	M2217_1.01	129	29	125	16	129	29	125	16	0.00E + 00	6.99E-01	−6.87E-02
M0769_1.01	M0769_1.01	43	10	20	17	43	10	20	17	0.00E + 00	6.09E-01	−1.52E-01
MYB1_binding_site motif	MYB1_binding_site motif	61	4	10	2	61	4	10	2	0.00E + 00	NA	NA
Gap-box_motif	Gap-box_motif	22	14	29	6	22	14	29	6	0.00E + 00	6.99E-01	−1.28E-01
Gap-box_motif	TELO-box_promoter motif	30	10	43	11	8	12	18	5	0.00E + 00	6.99E-01	2.59E-01
SBOXATRBCS	M0769_1.01	0	5	1	21	21	3	0	3	0.00E + 00	9.05E-01	1.25E-01
MYB1_binding_site motif	TELO-box_promoter motif	19	8	13	4	11	2	6	4	1.40E-03	6.99E-01	−2.65E-01
TELO-box_promoter motif	M0769_1.01	21	13	17	16	26	19	38	8	1.40E-03	7.65E-01	−5.71E-02
TELO-box_promoter motif	M1561_1.01	2	8	11	16	9	1	8	14	1.40E-03	6.99E-01	−3.64E-01
MYB3_binding_site motif	MYB3_binding_site motif	15	2	5	6	15	2	5	6	1.55E-03	8.56E-02	−1.02E + 00
Bellringer/replumless/pennywise_BS1_IN_AG	M2251_1.01	35	25	22	23	28	9	13	14	2.02E-03	6.99E-01	−9.38E-02
M0758_1.01	M0758_1.01	415	330	408	404	415	330	408	404	3.77E-03	1.45E-01	−9.02E-03
Bellringer/replumless/pennywise_BS1_IN_AG	M0758_1.01	274	318	313	287	341	245	272	322	4.65E-03	3.60E-01	7.94E-03
M1224_1.01	Gap-box_motif	9	3	7	11	9	4	19	6	1.62E-02	7.65E-01	1.83E-01
M0758_1.01	M1561_1.01	80	71	62	78	71	81	43	54	1.87E-02	6.99E-01	−5.21E-02
MYB3_binding_site motif	M0758_1.01	25	34	40	41	44	21	20	35	2.02E-02	3.88E-03	−2.17E-01

Motif pairs with pronounced non-random motif order and orientation preferences (motif-pair arrangement). Motif pairs were considered in both relative positions (motif 1 upstream of motif 2 or the reverse). For both arrangements, four different motif combinations of motif orientations are possible with “+”/”-“denoting forward and reverse-complement direction, respectively. P_BH_Entropy is the multiple-testing corrected *p*-value obtained from testing the significance of the motif order and orientation entropy (see Methods). P_BH_Expr is the *p*-value of the detected co-expression difference of genes associated with the most frequent arrangement relative to the three least frequent arrangements. Cohen’s d refers to the associated effect size with positive signs signifying increased co-expression among genes harboring the most frequent arrangement relative to the genes harboring the three least frequent arrangements. Motif pairs with 50 or more occurrences were considered only. Zero values of P_BH_Entropy indicate that the empirical *p*-value was below the limit of the shuffling repetitions (see Methods). For the MYB1_binding_site motif, insufficient gene expression information was available for the set of unique genes in which the pair was found. Note that for same-pair motifs, order is irrelevant and only four arrangements were considered

## Discussion

In this study, we investigated whether the orientation of cis-regulatory motifs in gene promoter regions relative to the transcriptional start site of downstream genes matters with regard to their effect on transcriptional regulation. To address this question, we pursued statistical approaches that exploit mapping statistics and co-expression analyses associated with 293 described cis-regulatory motifs in the plant species *Arabidopsis thaliana.* While positional intervals closer to the transcription start site (TSS) were found with increased frequencies of motifs exhibiting orientational preferences (Table 2, Fig. 3), an associated effect with regard to gene expression regulation as evidenced by increased co-expression of genes harboring the favored orientation in their upstream sequence could not be established (Table 2, Fig. 2). In fact, our results seem to even suggest a slight motif sequence selection against orientation-sensitivity, as true motif sequences passed our orientation filters at lower rates than what is randomly expected when assuming true motif composition alone (Fig. 2). Furthermore, we identified an intrinsic orientational asymmetry of sequence regions close to the TSS as the likely cause of the identified motif orientation preferences in close proximity to transcriptional start sites (TSS) (Fig. 4) (see below for further discussion of this point). Taken together and accepting the implemented test for co-expression as the deciding criterion, we did not find any convincing evidence in support of a critical role of motif orientation on the gene expression regulation of their respective downstream genes.

Naturally, it needs to be cautioned that “absence of evidence is not evidence of absence”. Therefore, we need to ask whether the implemented strategy and logic was reasonable and could have, in principle, revealed any orientation effect if present.

With regard to study design, as we were able to demonstrate that motif presence irrespective of orientation leads to significant statistical effects documenting motif activity with regard to gene expression regulation and thereby serving as a positive control (Table 2), we believe that the conducted co-expression test would have resulted in similar such evidence if motif orientation was important.

We based our conclusion on the percentage of actual motifs passing through various filtering steps in comparison to random controls and found no significant percentage differences. This does not mean, however, that individual motifs that were identified as orientation-sensitive are false-positives, but merely that for the set of motifs tested as a whole, no effect was discernable. Furthermore, even for motifs without general orientational preference across all its instances, individual genes and their regulation via promoter elements may very well depend on the correct orientation of such a motif as it may be possible that gene-specific additional factors impose constraints on the orientation of a motif in a particular genomic context that are not evident when probing for genome-wide preferences.

Hence, we also need to ask whether the set of 303 (including 10 core-promoter motifs) motifs as a whole may have been biased in favor of orientation-insensitive motifs. We collected motifs from two sources, 126 database/literature described motifs, whose details with regard to identification can be expected to be very diverse, and secondly, a consistent set of 176 motifs detected in a protein binding microarray (PGM) assay. For the latter, dependencies on genomic features has to be excluded as the binding to short sequences is probed. If anything, the set of 117 literature motifs may be enriched for direction-specific motifs. However, we did not find any difference in our results statistics comparing the two sets.

We observed increased fractions of motifs showing orientation mapping preferences when considering sequence intervals close to the TSS, in particular in the first upstream 100 nt (Fig. 3). This observation seems in line with the notion that constraints with regard to binding orientation of transcription factors and other proteins are more critical close to the TSS. However, as random motif sets also showed similar increases, we sought to explain this surprising observation and identified an underlying general sequence orientation asymmetry in sequence regions close to the TSS (Fig. 4). It needs to be cautioned that the cause-effect relationship may be unclear. As regions surrounding the TSS can be assumed enriched in cis-regulatory elements (including core-promoter elements), the ascribed general underlying sequence directionality may be the very consequence of motifs preferring a particular orientation. However, true motifs were found at lower frequencies to display orientation preferences than random motifs based on motif composition (Fig. 3). Thus, the observed orientation preference of true motifs is below random expectation implying that true motifs are even selected against orientation preferences. Only when assuming general base compositions and considering the first 200 nt upstream of the TSS, true motifs appear slightly elevated with regard to orientation preference relative to random motifs, albeit reaching significance in one interval (−200…−101 nt) only. By contrast, regions further upstream again suggest a weak selection of true motifs against positional preferences (Fig. 3). More importantly, the increased orientation preference near the TSS was not accompanied by a parallel effect with regard to gene expression, for which no significant differences compared to random expectations were detected (Table 2, Fig. 2). Thus, we are led to conclude that the observed motif orientation mapping statistics do not relate to any significant functional effects.

The nature of the observed sequence directionality in close proximity of the TSS (Fig. 4) remains to be investigated. Following observations that dinucleotide frequencies are non-random in RNAs caused by favorable base stacking interactions [51], dinucleotide shuffling procedures have also been implemented in the context of upstream-motif identification [3], but without explicit consideration of directional asymmetries. Barring any higher order sequence effects, forward and reverse-complement dinucleotide versions should indeed be indifferent with regard to physical parameters (stability, geometries etc.) as, chemically, the two versions – when present in a canonically base-paired double helix - are identical and differ only by a 180° rotation relative to the downstream TSS. Thus, it seems unlikely that orientation contributes to the discussed “physical code” around TSS [51]. Indeed, in a survey of bacterial and large viral genomes, dinucleotides were not found to display occurrence frequencies beyond the observed single-base strand compositional differences [52]. However, dinucleotide frequencies were assessed in large windows (50 kb) and not investigated specifically at distinctive genomic positions such as the region immediately upstream of the TSS as done in this study. Similarly, considering whole genome sequences, dinucleotide asymmetries were reported for selected genomes (human mitochondrial DNA), while generally, no dinucleotide strand biases were found [53]. Further studies on strand compositional differences – including plant genomes - did not address any higher order asymmetries beyond single base levels [47, 54, 55] or made no distinction between forward and reverse-complement orientation [51, 56]. Thus, either the observed strand asymmetries near TSSs are a consequence of the enriched orientation-specific motifs for which we, however, were not able to associate any functional relevance, or are shaped by additional physical or genomic constraints of as of yet unclear nature.

Our segmentation of upstream sequence intervals implied the location of the TSS to be known and to be unique per gene. As the correct determination of the TSS is not always guaranteed, and furthermore, multiple TSS sites may exist per gene [57], the assumption of a correctly positioned TSS cannot always be assumed true. However, as pronounced distance dependence effects were indeed identified in this study, and furthermore, sharply defined compositional changes at the surmised site of the TSS were determined (Additional file 2: Figure S1), taking the location of the TSS as reported in TAIR appears, in a statistical sense, reasonable.

Ideally, the issue of relevance of motif orientation would be resolved experimentally. However, current experimental techniques (ChipSeq, DNase-footprints) do not yield the required sequence resolution and typically range between 250–500 bp for ChipSeq. Novel technologies, e.g., Chip-exo [32], Chip-nexus [33], or X-Chip-Seq [34], may push the resolution limits for the obtained sequence lengths to allow for more direct motif identification. In addition to relying on increased resolution in pursuit of mapping statistics approaches, targeted interventions by flipping the orientation of selected candidate motifs would be even more desirable. Pursuing this avenue may be greatly facilitated by the recently emerging sequence editing techniques such as CRISPR-Cas9 [58].

On the technical side, we mapped motifs via their consensus sequence definitions that include ambiguity codes. Alternatively, motifs could also be mapped via their position specific weight matrix (PWM) representations [59]. However, as we needed to arrive at binary motif presence/absence calls, we considered both approaches similar, as in both cases, hard thresholds have to be introduced. In our methodology, this threshold is introduced at the level of motif definition, whereas PWM-based mapping procedures would require thresholds to be set for motif hit probabilities. Thus, in effect both methods, while not leading to identical results, are likely to lead to equivalent conclusions. In fact, we compared mapping results obtained for PWM-based motif definitions available for the set of 176 Cis-BP motifs to the mappings produced when using their respective consensus sequence definitions. (No PWM definitions were available from the respective databases for the remaining and largely literature based motifs.) Using the program fimo [59] and inspecting only those PWM-motifs that had identical lengths as our consensus motifs (where terminal Ns were cleaved off) and requiring more than 100 mapping sites detected in the 500 bp upstream regions, we found highly similar ratios of forward vs. reverse-complement motif hit counts when using consensus-sequence or PWM-motif definitions (Pearson correlation coefficient, *r* = 0.79, *p* = 4.8E-14, *N* = 59). Furthermore, the simpler consensus sequence mapping approach allowed us to easily create random motifs according to different background compositions and to compare them to actual motifs using the exact same mapping protocol. As the comparison to random motifs proved critical for the critical assessment of motif mapping statistics and downstream effects, and furthermore, not for all motifs PWMs were available, we decided in favor of the simpler consensus motif mapping approach. Motifs can also be defined as Hidden Markov Models (HMM), which, in addition to single position base variation, also capture dependencies between positions within a motif [60–62]. However, building HMM-models requires a set of true positive motif variants associated for a given transcription factor. As both consensus and PWM-based motif mappings will inevitably produce false-positive hits, a reliable true positive set is best obtained experimentally. However, given the current resolution of existing experimental methods (see above), it is not yet possible to clearly identify, which motif is actually represented by the sequence region identified to be occupied by a transcription factor and large numbers of observations are necessary to establish an enrichment of candidate motif in the sequenced footprints. Efforts to build HMMs from protein binding microarray datasets that provide protein binding information to very short sequences (8–10 nt) [12, 63], or from phylogenetic information [64] may prove useful. In addition to more refined motif mapping algorithms, sequence conservation across different species or individuals within a given species (single nucleotide polymorphisms (SNP)) may help to identify true positive motifs. However, SNP-densities need to be very high to allow for a reliable detection of local conservation [20]. Motifs can also be species-specific and furthermore are not guaranteed to reside at similar distances relative to the TSS rendering their identification difficult. Irrespective of mapping and motif identification procedures, it is clear that false-positive motif mapping sites will be generated. Thus, our study draws its validity from the statistics of a large number of observations.

Taken together, we believe that our study would have revealed any significance of motif orientation if present. Thus, we conclude that the orientation of cis-regulatory motifs in gene promoter regions generally does not matter with regard to transcriptional regulation of downstream genes in *Arabidopsis thaliana.* This would imply that rather than conformational details and precise positioning of proteins involved in the triggering of transcription, the event of binding itself may constitute the relevant regulatory event. Transcription factor binding may lead to local modifications of the DNA structure (bending, melting etc.) required to initiate transcription. For example, it was reported that local structural changes indeed lead to transcriptional regulatory effects [65]. Orientation-effects have also been shown to be associated with chromatin remodelers [66] suggesting that nucleosome-associated processes may also be relevant in determining transcriptional directionalities.

Cis-regulatory motifs are typically reported as short sequence motifs for which specificity of transcription factor binding appears difficult to ensure. Indeed, recently it was shown that the sequence context of motifs plays critical role in rendering motifs active or inactive [67]. Thus, with regard to orientational effects, sequence context may need to be examined explicitly in possible extensions of this study.

Despite identifying positional and relative orientational preferences of motif pairs, no relevance with regard to gene expression regulation was detected. In the case of motif order (position within the upstream region), preferred arrangements may therefore simply reflect tendencies of individual motifs to be positioned at closer or farther distances relative to the TSS. For motif pairs to be functional, the distance between motifs in a pair can be assumed relevant [17]. However, when repeating the pair analyses considering only motif pairs separated by less than 100 bp with an assumed tight interaction, no motif pair reached significance with regard to motif order preferences.

We conducted our analysis in a eukaryotic plant system (*Arabidopsis thaliana*). Evidently, the analysis can be expanded to other well characterized model prokaryotic and eukaryotic organisms such as E.coli, yeast, or human.

In the introduction, we illustrated the effect of motif orientation on the spatial alignment of transcription factors relative to its downstream gene. Typically, transcription factors will not possess a 180°-rotational symmetry, in which case motif orientation would not matter at all. One conceivable way to create rotation-symmetric molecules is by homo-dimerization. Indeed, transcription factors are frequently active as dimers, both homo- and heterodimers [1, 4, 68]. If acting as a homodimer, regardless of motif orientation, the orientation of the transcription factor dimer would be invariant as one half of the dimeric transcription factor would find its target sequence present either in forward or reverse-complemented orientation. However, a second instance of the motif in the reverse-complement orientation needs to be present as the second monomer may also need to bind to DNA thereby creating a palindromic motif. Casting doubt on this scenario, we did not find elevated frequencies of antiparallel orientations of same-motif pairs (Fig. 5, central panel). Alternatively, only one binding interface would be sufficient for binding with the second monomer binding un-specifically. Investigating this possible binding mode pursuing statistical approaches (reported homodimers, neighboring motif occurrences) therefore appears worthwhile.

Alternatively, orientation indifference may already be built into the TFBS itself. Palindromic motifs are identical in sequence when reversed and complemented. In the original set of 323 unique motifs with four or more none-N bases (including data sources A and B, see Methods), 30 (9.3 %) were palindromic. (Note that palindromic motifs were excluded from further analyses because of their built-in orientation-invariance.) Among a large set of randomly generated motifs following the general motif composition and with the same length distribution as the 323 original motifs, only 0.08 % were palindromic (11 out of 12,920 (=40 times the size of the original set size). Thus, real motifs are significantly enriched in palindromic motifs (*p* = 1.1E-50 in a binomial distribution test). This further supports our conclusion that evolution has acted in favor of motif-orientation indifference.

## Conclusions

Transcription initiation and regulation is a complex process with many additional relevant factors that need to be considered such as enhancer elements, nucleosome organization, DNA methylation, and many more. Here, we focused on the question of the relevance of cis-regulatory motif orientation in gene upstream promoter regions. No evidence was found for motif orientations to be preferred across all motif instances and which can be associated with detectable, regulatory gene expression effects. We conclude that, generally, motif orientation effects either do not play a significant role in the regulation of gene expression in the plant *Arabidopsis thaliana* or are revealed only at the level of particular loci in conjunction with gene-specific additional factors in need of targeted experimental analyses.

## Methods

### Cis-regulatory transcription factor binding site motifs

Cis-regulatory motifs reported in *Arabidopsis thaliana* were collected from three sources. A) As used in [20], a set of 137 literature-described motifs and aggregated from three different databases resources AGRIS [69], Athena [70], and PLACE [71]. B) A set of 297 Arabidopsis motifs detected in protein binding microarray (PBM) profiling experiments [3] and obtained from the Cis-BP database, Version 1.01 (http://cisbp.ccbr.utoronto.ca/). C) A set of 10 core promoter motifs reported in [46] as enriched in the 50 bp upstream sequence interval of Arabidopsis genes. For set C, consensus motifs were created based on the provided sequence logos in [46]. This set included the TATA-box motif that is contained in set A as well. In set C, we used the sequence definition as reported in the literature as “TATAAA” (set A).

As consensus motif sequences frequently contain ambiguous bases resulting in reduced specificity, all motifs were required to possess at least four unambiguous bases (A, C, G, or T) to ensure a minimal sequence mapping specificity. All palindromic motifs and motifs found identical to other motifs in the combined set were removed. Further filtering identified a) motifs that were found fully contained in longer motifs based on their explicit definition; i.e., not considering all possible sequence variants associated with ambiguous bases, and b) by considering all sequence variants defined by ambiguous bases in the respective motifs. As motifs with consecutive “N”-bases cause all motifs of this length of shorter to be eliminated as contained in larger motifs, motifs with 4Ns or more were eliminated (removing two motifs). Motifs found contained in longer motifs were kept, but marked. Ambiguous terminal bases signified by the character “N”, i.e., any of the four canonical bases, as found in Cis-BP motif definitions were removed and motifs truncated accordingly.

In total, a set of 303 *Arabidopsis thaliana* cis-regulatory motifs (avg. length 9.72 bp) was used in this study with 117 obtained from literature-reported motifs (source A) and 176 originating from the Cis-BP dataset (source B), and 10 core promoter motifs (source C). The more stringent filtering step b) reduced the number of unique motifs to 62 motifs (not considering the core-promoter motifs). This motif set was used for the analysis of motif pairs. The set of core promoter motifs was analyzed separately and mapped to the 50 bp upstream regional intervals only. All motifs and their sequence definitions are contained in Additional file 1.

### Upstream sequences

Genomic sequences of length 500 bp upstream of all annotated 33,323 nuclear-encoded Arabidopsis genes were downloaded from TAIR, version 10 [72]. All sequences correspond to coding-strand sequences according to the identified orientation of the downstream gene. To specifically investigate upstream regions in closer proximity to the transcriptional start site (TSS) of genes, sequences of length 250 bp and 100 bp, respectively, as well as non-overlapping intervals of length 100 bp were excised and analyzed separately. Mitchondrial and chloroplastidial sequences were not considered.

### Randomized motif sequences

To serve as controls, motif mapping statistics and expression effect analyses were repeated for randomized versions of all motifs used in this study. Random motifs were created to follow exactly the same length distribution as observed in the actual motif set; i.e., every motif was replaced by a random motif of identical length. Motif positions were filled with bases drawn from two distinct background base compositional distributions: A) base frequencies as observed in the set of motifs used in this study (including ambiguity codes), and B) base frequencies as determined in the respective upstream sequences of Arabidopsis genes (listed in Table 1). To ensure statistical robustness, five times as many random motifs were created than the number of true motifs; i.e., 5x293 true motifs, 5x10 core promoter elements. Comparisons of random motif mappings (Additional file 2: Table S1) to the mapping statistics obtained for true motifs (Table 2) were based on the two-sided Fisher exact test.

### Mapping procedure

Motifs were mapped to upstream sequences using the Perl-programming language string matching function. Ambiguous bases represented by the respective IUPAC ambiguity codes were replaced by the set of associated canonical bases A, C, G, or T, and string matching was done using regular expressions allowing for the specified variability at a given position. The Perl-string matching procedures resulted in all non-overlapping motif matches in a given sequence. Motifs were mapped taking the motif-sequence definition as reported in the resource it was derived from as well as in the generated reverse-complemented version to the upstream coding strand region of lengths 500 bp, 250 bp, or 100 bp, respectively, as well as to the (−500 bp,−50 bp) interval and non-overlapping intervals of length 100 bp covering all 500 bp usptream.

### Mapping orientation preference

Motifs were checked for evidence of preferred mapping orientation (forward or reverse-complement) based on binomial distribution tests by comparing the fraction of observed forward mappings of a given motif to its expected random fraction of forward mappings based on the composition of upstream regional intervals. The latter was estimated from Eq. 1 as:

1 $$ {F}_{fw,m}=\frac{P_{fw,m}}{P_{fw,m}+{P}_{rc,m}}\  with\ {P}_{fw/rc,m}={\displaystyle {\prod}_i}{p}_{base,\ fw/rc}(i), $$

where p_base,fw/rc_(i) corresponds to the probability of observing the particular base at position i in motif m in its forward (fw) or reverse-complement (rc) sequence definition based on the observed relative frequency of this base in the considered upstream region (Table 1). In case of ambiguously defined bases, probabilities were summed up for the correspondingly allowed base types. Actual orientation preference was determined as the sign of the logarithmic ratio of counts of actual forward vs. reverse-complement mappings relative to the ratio obtained from P_fw/_P_rc_.

### Positional preferences of motifs, Positional Entropy (PE)

To check for possible orientation-dependent positional preferences in the upstream sequences relative to the translational start site, statistics of mapping locations were assessed employing the concept of entropy. Positional entropies, PE, were computed for all motifs, m, according to

2 $$ P{E}_m={\displaystyle {\sum}_i}{p}_i log\left({p}_i\right), $$

where i corresponds to a positional interval, and p_i_ is the relative frequency of observing motif m in interval i, the log was taken relative to base e. Upstream sequences were partitioned into 10 equally sized, non-overlapping intervals. Upstream sequences of length 500 bp only were analyzed as for shorter sequences, positional preferences lose their meaning as they are by definition confined to a small interval. Motifs with large PE will be distributed relatively evenly across the upstream segments, whereas motifs with small PE will tend to be confined to specific positional intervals.

### Motif- and motif-orientation-specific gene sets for comparative gene expression analysis

To assess the effect of motif presence vs. absence on the regulation of the respective downstream genes, two gene sets were generated for the respective motif and submitted to gene expression correlation analysis as detailed below. Motif presence or absence was defined in two ways. For assessing the effect of the actual presence of a given motif in the considered upstream region irrespective of motif orientation (forward or reverse-complement), all genes harboring the motif in their upstream regions of a given length were taken as the positive set and compared to a set of genes not containing the respective motif (negative set). The negative set was chosen randomly from the set of all Arabidopsis genes not containing the motif such that the set was comparable in size to the positive set, but were selected such to contain at least 100 genes. This sampling was implemented to prevent prohibitively large negative sets – typically motifs are found in only few genes leaving tens of thousands of genes in the negative set – and to guarantee a minimum size in cases when the motif is found in only very few genes. Orientation effects were assessed similarly, whereby gene sets were compared whose upstream sequences contained a given motif in the forward direction, and in the forward direction only, to those genes harboring the same motif in the reverse-complement orientation, and in the reverse-complement direction only. Enforcing set size limits was not necessary for the latter comparison as actual set sizes allowed for an efficient computation and no stark asymmetries of the positive versus the negative set size were present.

### Assessment of motif presence/absence effect as judged by gene co-expression analysis

Motifs were tested for evidence of functional relevance based on gene co-expression analysis following the same protocol as introduced in [20]. Two gene sets as described above were compared, in which one set contained genes harboring the motif, whereas the other does not. Motif presence and absence was understood either as actual presence vs. absence of the motif in the upstream sequence regardless of orientation, or as either the motif being present in forward as opposed to reverse-complement orientation with the corresponding the genes combined in the second set.

In brief, the co-expression analysis protocol uses a large set of 5295 gene expression profiling experiment based on the ATH1-Affymetrix gene-chip platform containing 20,922 Arabidopsis gene transcripts with unique chip probes to gene identifier mappings. Based on normalized and log-transformed expression values, pairwise Pearson correlation coefficients between expression values across all experiments were computed for all possible gene-pairs in a set. Differences between two sets; i.e., higher or lower correlation within genes in one set versus the other, were judged based on Wilcoxon rank-sum tests applied to the computed correlation coefficients (p_r_diff_) and the magnitude of the difference assessed by Cohen’s d, a measure of effect size [73]. Essentially, Cohen’s d compares the difference of the mean coefficients to the average standard deviation in the two sets. For a detailed description, see [20].

### Dinucleotide orientational asymmetries in gene upstream regions

Evidence of sequence directionality in gene upstream regions was assessed at the level of dinucleotides. For every of the 16 possible dinucleotides associated with the four canonical bases (B) A, C, G, and T, occurrence ratios, R, comparing their observed relative frequencies, f_obs_, in a given sequence interval to their expected probability estimated from the observed base composition in the same interval and assuming positional independence were computed according to:

3 $$ {R}_{B1B2}=\frac{f_{B1B2}}{p_{B1}*{p}_{B2}}, $$

with f_B1B2_ denoting the observed relative frequency of dinucleotide B1B2, and p_B1_/p_B2_ estimating the probability of bases B1 and B2, respectively, estimated from the observed relative base frequencies in the respective sequence interval. Dinucleotide orientation ratios (DORs) were then computed by comparing occurrence ratios of a given dinucleotide in forward orientation to its respective ratio obtained assuming its reverse-complement with:

4 $$ DO{R}_{B1B2/rc(B1B2)}=\frac{R_{B1B2}}{R_{rc(B1B2)}}, $$

with rc() denoting reverse-complement. Note that for palindromic dinucleotides (AT, TA, CG, GC), DORs compute as one.

### Motif pair statistics

Enrichment of co-occurrence of motifs in upstream regions of the same genes was assessed applying the hypergeometric distribution. Only non-overlapping (mapping position) motif combinations were considered as valid instances of co-occurring motifs; i.e., an upstream region was declared to contain the two motifs in question, when they were found with non-overlapping hits.

Preferences for specific relative sequence positions; i.e., the order of motifs in a pair, and orientation (forward, reverse-complement) was tested for all motif pair instances found in the same upstream region. For any given motif pair, eight different combinations are possible (motif order (motif1/2 followed downstream by motif 2/1) with respective forward (fw) and reverse-complement (rc) orientations: fw-fw, fw-rc, rc-rc, rc-fw). To detect significant deviations from random expectation of occurrence frequencies across all eight possible motif combinations by a single test, we employed the concept of entropy defined by Eq. 2. Here, the probabilities, p_i,_ correspond to the relative frequencies of individual arrangements (e.g., relative frequency of motif 1 in forward orientation followed by motif 2 in reverse-complement orientation, likewise for all other seven arrangements) with the summation over all eight possible motif pair arrangements. To avoid zero-counts and associated error when taking the logarithm, “1” was added to all eight arrangements. Statistical significance was assessed by computing empirical p-values derived from entropy value distributions for 100,000 random arrangements considering the total number of occurrences of a given motif pair and with the empirical *p*-value taken as the fraction of random arrangements yielding smaller entropy values than obtained for the actual arrangement counts. Note that the random distribution is not distributed normally. In a second step, this procedure was repeated for the four motif combinations in which a given motif 1 precedes a given motif 2, followed by a separate assessment of the four combinations with the reverse motif order. This step was implemented to detect orientation-sensitive motif combinations as opposed to motif pairs that exhibit a preferred motif order only, which would also be detected in the first passage testing all eight combinations. In addition to the entropy-based test metric, tests for preferred sequential motif order were performed based on the binomial test assuming an expected chance of 50 % for both possible orders.

The relevance of detected preferred motif pair arrangements was tested based on evidence of increased levels of co-expression (as explained above) for genes with a particular motif arrangement that was found preferred relative to genes harboring the same motif pair, but in alternative combinations.

### General statistics

All statistical computations were performed using R. Significance *p*-values were corrected for multiple testing whenever necessary applying the Benjamin-Hochberg method [48].

## Additional files

Additional file 1: Excel document containing comprehensive motif, motif mapping, and co-expression correlation statistics information. (XLSX 242 kb) Additional file 2: Figure S1. Compositional skew in gene upstream regions of length 500nt in *Arabidopsis thaliana*. Plotted are the skew ratios of the frequencies of canonical base types, G and C, and, A and T, respectively, where the letters denote the identity of the bases and their respective relative frequency. Ratios were computed for every sequence position separately. The graphs reveal a bias towards increased occurrences of C relative to G as well as an increased frequency of A relative to T near the transcription start site (TSS). Peaks observed at positions -25nt likely correspond to the TATA-box motif, and near zero, to the distinct sequence compositions at the TSS. **Table S1.** Motif mapping and co-expression analysis results for random motif sets. Random motifs were created based on R1) the reference composition observed in the respective upstream sequence interval or R2) the composition of true motifs, and with motif lengths according to the length of the 293 true/ 10 core promoter motifs. Large sets of 5x293 random motifs and 5x10 random core promoter motifs were generated with results reported for all random motifs yielding valid results (sufficient mapping statistics, available gene expression information). Table columns as in Table 2 (main text). random motifs was generated with results reported for all random motifs yielding valid results (sufficient mapping statistics, available gene expression information). *P*-values in columns C-F denote the significance of deviation of the random control set relative to the actual motif set based on Fisher’s exact test (Table 2) with indicating both the raw *p*-value and the Benjamini-Hochberg corrected *p*-value (BH) considering the five different intervals for which each particular test (e.g., D2) was performed and considering each randomization type separately. *P*-values are underlined if *p* < 0.05. Font colors indicate higher (red) or lower (blue) percentages than observed for true motifs irrespective of significance. (PDF 130 kb)

## Abbreviations

bp: base pair
nt: nucleotide
TF: transcription factor
TFBS: transcription factor binding site
TSS: transcription start site
