# Erratum to: ‘The orientation of transcription factor binding site motifs in gene promoter regions: does it matter?’

**DOI:** 10.1186/s12864-016-2630-5

**Published:** 2016-04-28

**Authors:** Monika Lis, Dirk Walther

**Affiliations:** Max Planck Institute for Molecular Plant Physiology, Am Mühlenberg 1, 14476 Potsdam-Golm, Germany

Unfortunately, the original version of this article [1] contained an error. Equation number two was missing a “–“ (a minus) sign before the sum symbol. The correct equation has been included here. This correction concerns the notation only. All computations using this equation were done correctly.

2 $$ P{E}_m=-{\displaystyle {\sum}_i{p}_i log\left({p}_i\right)}, $$

## References

[1] Lis M, Walther D (2016). The orientation of transcription factor binding site motifs in gene promoter regions: does it matter?. BMC Genomics.

