# Supplementary material for: The orientation of transcription factor binding site motifs in gene promoter regions: does it matter?
Source: BMC Genomics. 2016 Mar 3;17:185. doi: 10.1186/s12864-016-2549-x (PMC4778318; doi:10.1186/s12864-016-2549-x)
Supplement: Additional file 2: Figure S1. — Compositional skew in gene upstream regions of length 500nt in Arabidopsis thaliana. Plotted are the skew ratios of the frequencies of canonical base types, G and C, and, A and T, respectively, where the letters denote the identity of the bases and their respective relative frequency. Ratios were computed for every sequence position separately. The graphs reveal a bias towards increased occurrences of C relative to G as well as an increased frequency of A relative to T near the transcription start site (TSS). Peaks observed at positions -25nt likely correspond to the TATA-box motif, and near zero, to the distinct sequence compositions at the TSS. Table S1. Motif mapping and co-expression analysis results for random motif sets. Random motifs were created based on R1) the reference composition observed in the respective upstream sequence interval or R2) the composition of true motifs, and with motif lengths according to the length of the 293 true/ 10 core promoter motifs. Large sets of 5x293 random motifs and 5x10 random core promoter motifs were generated with results reported for all random motifs yielding valid results (sufficient mapping statistics, available gene expression information). Table columns as in Table 2 (main text). random motifs was generated with results reported for all random motifs yielding valid results (sufficient mapping statistics, available gene expression information). P-values in columns C-F denote the significance of deviation of the random control set relative to the actual motif set based on Fisher’s exact test (Table 2) with indicating both the raw p-value and the Benjamini-Hochberg corrected p-value (BH) considering the five different intervals for which each particular test (e.g., D2) was performed and considering each randomization type separately. P-values are underlined if p < 0.05. Font colors indicate higher (red) or lower (blue) percentages than observed for true motifs irrespective of significance. (PDF 130 kb) [file 12864_2016_2549_MOESM2_ESM.pdf]

The orientation of transcription factor binding site motifs in gene promoter regions: does it matter?

Monika Lis and Dirk Walther

Additional File 2

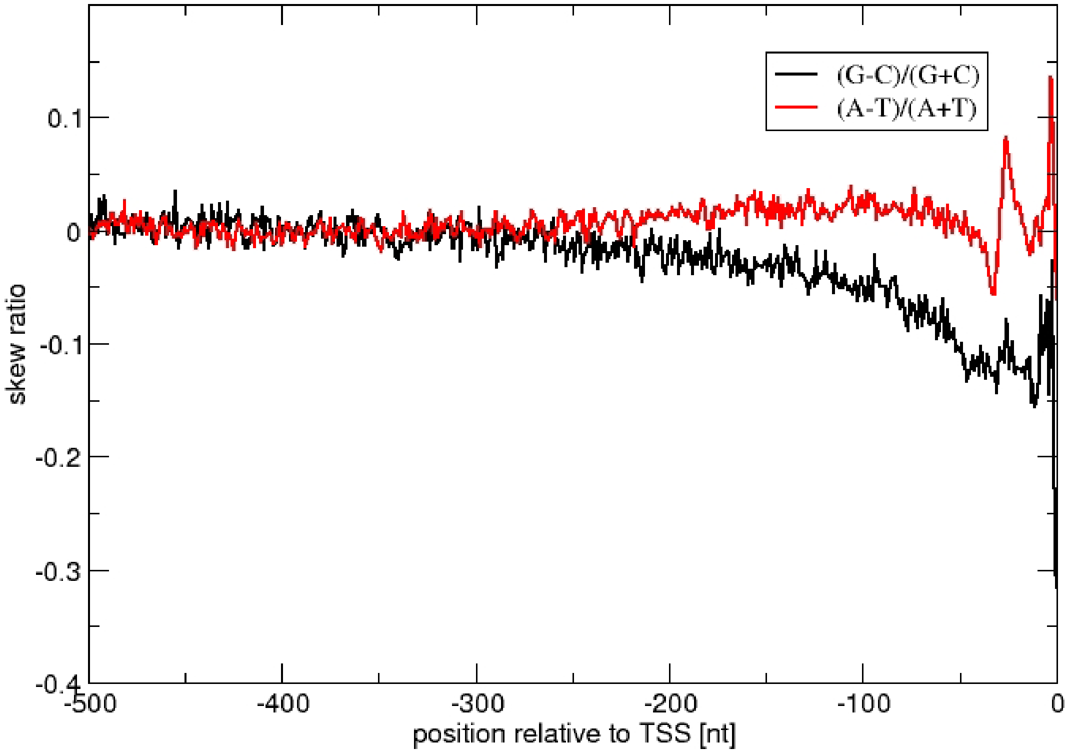

Supplementary Figure S1 Compositional skew in gene upstream regions of length 500nt in *Arabidopsis thaliana*. Plotted are the skew ratios of the frequencies of canonical base types, G and C, and, A and T, respectively, where the letters denote the identity of the bases and their respective relative frequency. Ratios were computed for every sequence position separately. The graphs reveal a bias towards increased occurrences of C relative to G as well as an increased frequency of A relative to T near the transcription start site (TSS). Peaks observed at positions -25nt likely correspond to the TATA-box motif, and near zero, to the distinct sequence compositions at the TSS.

Random motif mapping and co-expression statistics

A) Up-stream sequence interval [bp]	B) Number of motifs	C) Presence/ absence statistic, p-value relative to true motifs , BH-corrected p-value	D) Mapping orientation statistic, D1/D2/D3 p-value relative to true motifs, BH-corrected p-value	E) Orientation and presence/ absence effect (% of C), p-value relative to true motifs , BH-corrected p-value	F) Set E with PE-filter (% of E), p-value relative to true motifs
**R1) Randomization based on respective upstream sequence interval base composition**					
-500 , -1	1147	203 (17.7%, p=1.9E-16, 3.1E-16)	158 (13.7%, p=0.69, 0.86)/ 65 (5.7%, p=0.012, 0.06)/ 44 (3.8%, p=0.13)	10 (4.9%. p=1, 1)	6 (60%, p=0.34)
-250, -1	1102	228 (20.7%, p=8.9E-22, 4.4E-21)	223 (20.2%, p=0.13, 0.57)/ 87 (7.9%, p=0.038, 0.095)/ NA	23 (10.1%, p=1, 1)	NA
-100, -1	1032	214 (20.7%, p=3.1E-17, 7.7E-17)	215 (20.8%, p=0.44, 0.73)/ 72 (6.9%, p=0.22, 0.27)/ NA	22 (10.3%, p=0.57, 0.95)	NA
-500, -51	1127	171 (15.2%, p=1.1E-15, 1.4E-15)	125 (11.1%, p=1,1)/ 49 (4.3%, p=0.1, 0.17)/ NA	2 (1.1%, p=0.1, 0.5)	NA
-50, -1 (core motifs)	20	2 (10%, p=0.007, 0007)	5 (25%, p=0.23, 0.57)/ 1 (5%, p=1, 1)/ NA	1 (50%, p=0.25, 0.62)	NA
**R2) Randomization based on base composition of true motifs**					
-500 , -1	1193	285 (23.9%, p=4.4E-09, 7.3E-09)	271 (22.7%, p=0.004, 0.015)/ 131 (11.0%, p=0.83, 1)/ 87 (7.3%, p=0.59)	26 (9.1%, p=0.42, 0.6)	20 (76.9%, p=1)
-250, -1	1180	292 (25.5%, p=3.9E-16, 9.7E-16)	340 (28.8%, p=0.2, 0.25)/ 178 (15.1%, p=0.25, 0.62)/ NA	40 (13.7%, p=0.27, 0.6)	NA
-100, -1	1144	298 (26.0%, p=1.3E-19, 6.4E-19)	367 (32.1%, p=0.006, 0.015)/ 175 (15.3%, p=0.015, 0.075)/ NA	51 (17.1%, p=0.012, 0.06)	NA
-500, -51	1193	265 (22.2%, p=1.5E-07, 1.9E-07)	174 (14.6%, p=0.11, 0.18)/ 78 (6.5%, p=0.78, 1)/ NA	12 (4.5%, p=0.78, 0.78)	NA
-50, -1 (core motifs)	42	7 (16.6%, p=0.01, 0.01)	14 (33.3%, p=0.47, 0.47)/ 4 (9.5%, p=1, 1)/ NA	2 (28.6%, p=0.46, 0.6)	NA

Supplementary Table 1. Motif mapping and co-expression analysis results for random motif sets. Random motifs were created based on R1) the reference composition observed in the respective upstream sequence interval or R2) the composition of true motifs, and with motif lengths according to the length of the 293 true/ 10 core promoter motifs. Large sets of 5x293 random motifs and 5x10 random core promoter motifs were generated with results reported for all random motifs yielding valid results (sufficient mapping statistics, available gene expression information). Table columns list

information on A) the interval of the considered upstream regions, B) the number of considered motifs with valid observations, C) Number (percentage) of motifs with significant co-expression differences between genes containing the genes upstream regardless of direction compared to genes not containing the motif at all (neither in forward nor reverse-complement orientation) with thresholds $p_{r\_diff}<0.05$ and Cohen's $d>0.01$. D) Motif mapping statistics with D1 indicating the number of motifs with significant orientation preference ($p_{orient}<0.05$), D2 - subset of D1 meeting also the criteria of significant co-expression differences ($p_{r\_diff}<0.05$) with higher intra-set correlations in the set corresponding to the preferred mapping orientation, and, in addition (D3), lowered positional entropy (PE) in the preferred orientation. As no positional entropies were computed for the shorter upstream intervals of length 250bp and 100bp, D3 is not provided for those sets. E) Filter criteria D2 applied only to the subset of motifs with evidence of significant presence/absence effect (column B) (Note that the multiple testing correction was adjusted accordingly.) F) Subset of E that also exhibit lowered positional entropy (PE) in the preferred orientation (Filter criteria D3, applied to upstream regions of length 500bp only as positional preferences lose their meaning for smaller considered sequence intervals). Random motifs were created based on the reference composition observed in the respective upstream sequence interval with motif lengths according to the length of the 293 actual motifs. A large set of 5x293 random motifs was generated with results reported for all random motifs yielding valid results (sufficient mapping statistics, available gene expression information). P-values in columns C-F denote the significance of deviation of the random control set relative to the actual motif set based on Fisher's exact test (Table 2) with indicating both the raw p-value and the Benjamini-Hochberg corrected p-value (BH) considering the five different intervals for which each particular test (e.g. D2) was performed and considering each randomization type separately. P-values are underlined if $p<0.05$. Font colors indicate higher (red) or lower (blue) percentages than observed for true motifs irrespective of significance.
